# Peripheral blood gene expression patterns discriminate among chronic inflammatory diseases and healthy controls and identify novel targets

**DOI:** 10.1186/1755-8794-3-15

**Published:** 2010-05-05

**Authors:** Bertalan Mesko, Szilard Poliska1, Andrea Szegedi, Zoltan Szekanecz, Karoly Palatka, Maria Papp, Laszlo Nagy

**Affiliations:** 1 Department of Biochemistry and Molecular Biology, University of Debrecen, Debrecen, Hungary; 2 Apoptosis and Genomics Research Group of the Hungarian Academy of Sciences, Research Center for Molecular Medicine, Medical and Health Science Center, University of Debrecen, Debrecen, Hungary; 3 Department of Dermatology, Medical and Health Science Center, University of Debrecen, Debrecen, Hungary; 4 Clinical Genomics Center, Medical and Health Science Center, University of Debrecen, Debrecen, Hungary; 5 Department of Rheumatology, Institute of Medicine, University of Debrecen, Medical and Health Science Center, Debrecen, Hungary; 6 2nd Department of Medicine, University of Debrecen, Medical and Health Science Center, Debrecen, Hungary

## Abstract

**Background:**

Chronic inflammatory diseases including inflammatory bowel disease (IBD; Crohn's disease and ulcerative colitis), psoriasis and rheumatoid arthritis (RA) afflict millions of people worldwide, but their pathogenesis is still not well understood.

It is also not well known if distinct changes in gene expression characterize these diseases and if these patterns can discriminate between diseased and control patients and/or stratify the disease. The main focus of our work was the identification of novel markers that overlap among the 3 diseases or discriminate them from each other.

**Methods:**

Diseased (n = 13, n = 15 and n = 12 in IBD, psoriasis and RA respectively) and healthy patients (n = 18) were recruited based on strict inclusion and exclusion criteria; peripheral blood samples were collected by clinicians (30 ml) in Venous Blood Vacuum Collection Tubes containing EDTA and peripheral blood mononuclear cells were separated by Ficoll gradient centrifugation. RNA was extracted using Trizol reagent. Gene expression data was obtained using TaqMan Low Density Array (TLDA) containing 96 genes that were selected by an algorithm and the statistical analyses were performed in Prism by using non-parametric Mann-Whitney U test (P-values < 0.05).

**Results:**

Here we show that using a panel of 96 disease associated genes and measuring mRNA expression levels in peripheral blood derived mononuclear cells; we could identify disease-specific gene panels that separate each disease from healthy controls. In addition, a panel of five genes such as ADM, AQP9, CXCL2, IL10 and NAMPT discriminates between all samples from patients with chronic inflammation and healthy controls. We also found genes that stratify the diseases and separate different subtypes or different states of prognosis in each condition.

**Conclusions:**

These findings and the identification of five universal markers of chronic inflammation suggest that these diseases have a common background in pathomechanism, but still can be separated by peripheral blood gene expression. Importantly, the identified genes can be associated with overlapping biological processes including changed inflammatory response. Gene panels based on such markers can play a major role in the development of personalized medicine, in monitoring disease progression and can lead to the identification of new potential drug targets in chronic inflammation.

## Background

Chronic inflammatory diseases such as inflammatory bowel disease (IBD; including Crohn's disease - CD and ulcerative colitis - UC), psoriasis and rheumatoid arthritis (RA) exist as a substantial burden in social and economic terms worldwide. Despite the importance of these diseases, it is still not clear if characteristic gene expression signatures can discriminate this group of diseases from healthy controls, the various diseases from each other or whether it is possible to stratify the diseases based on gene expression changes.

These chronic conditions have common features such as the autoimmune origin, the frequent co-morbidity and a few genes such as IL10, IL23R, SLC22A4 and SLC22A5 that have been identified as contributors to their genetic background [Table 1]. However their prevalence and the tissues affected are clearly different.

**Table 1:** Known SNP - disease associations


**Gene**	**IBD**	**Psoriasis**	**Rheumatoid arthritis**

ADAM33	NA	rs512625 PMID: 18560587	**NA**

IL10	**rs3024505 PMID: 18836448**	**NA**	**rs1800896 PMID: 18615156**

IL13	NA	**rs1800925 PMID: 19554022**	NA

IL23R	rs2201841 PMID: 18338763	rs11209026 PMID: 18369459	**NA**

IL4	**rs2243250 PMID: 18064451**	NA	NA

IL8	NA	NA	**PMID: 18799095**

PADI4	**NA**	**NA**	rs2240340 PMID: 12833157

PTGS2	rs20432 PMID: 16273614	**NA**	**rs5275 PMID: 18381795**

PTPN22	NA	rs1217414 PMID: 18341666	**rs2476601 PMID: 18466513**

SLC22A4	**rs3792876 PMID: 17476680**	rs11568506 PMID: 18614543	rs3792876 PMID: 15107849

SLC22A5	rs3792876 PMID: 17476680	rs2631367 PMID: 16255050	**rs2631367 PMID: 15107849**

Known associations between single nucleotide polymorphisms and chronic inflammatory diseases are shown in the table with SNP ID and PMID number referring to the publication that documents the association. NA means there is no documented association on SNP level. Bold cells mean we could detect the association in our study between that specific disease and healthy controls.

RA is a systemic autoimmune disorder, with a prevalence between 0.5-1.0% [1], that causes inflammation and tissue damage in joints and tendon sheaths. Psoriasis which is a chronic disorder of the skin and joints where the psoriatic plaques are areas of inflammation and excessive skin production, affects approximately 2% of the population only in the USA [2]. 1-2% of Western populations suffer from IBD [3] in which the common features are the inflammation of the intestines and the autoimmune origin.

Global and selective gene expression analyses have already been performed in order to gather hints on the mechanisms of these medical conditions by using human biopsy samples such as colon tissue in IBD [4]; skin tissue in psoriasis [5] and synovial tissue biopsy in RA [6]. However peripheral blood is a more accessible source of cells and may be easier to use for screening processes. Furthermore as circulating peripheral blood mononuclear cells (PBMCs) are key cells of inflammation, it may also reflect disease mechanisms.

In addition studies of gene expression profiling of PMBCs may provide a more cost effective and less invasive alternative to biopsy or invasive measurements [7]. Examples of the clinical implications of this approach include the analysis of human breast cancer progression [8] and PBMC profiles in RA, systemic lupus erythematosus, type I diabetes and multiple sclerosis [9].

It appears therefore that gene expression profiling from PBMCs is a validated tool for discovery and also may be used to explore the pathogenetic background of these medical conditions [10-12]. However comparative studies on the existence of distinct and overlapping gene expression patterns are lacking. We sought to fill this gap by carrying out a comparative analysis of peripheral gene expression patterns of a panel containing 96 genes in various chronic inflammatory diseases and healthy controls.

## Methods

### Patient recruitment

The Research Ethics Committee of University of Debrecen Medical and Health Science Center approved the clinical protocol and the study that were in compliance with the Helsinki Declaration. Signed informed consent was obtained from all healthy and diseased individuals who provided blood sample. Inclusion and exclusion criteria were developed using the best evidence currently available. Online supplement is provided for additional information about inclusion and exclusion criteria [Additional File 1: Figure S2].

The study included 13 patients with IBD; 15 with psoriasis and 12 with RA, all of whom had active disease and were medication-free at the time of blood draw [Table 2]. Blood was also obtained from a group of healthy control individuals (18 patients) that did not show significant differences compared to the diseased groups regarding age. After the subjects fasted overnight for 12 hours, all of the blood samples were obtained locally between 8:00 AM and 9:00 AM; and were processed within one hour after sample collection.

**Table 2:** Patient parameters

Disease Status	Control	IBD		Psoriasis	Rheumatoid arthritis
n	18	13		15	12

Sex (male/female)	8/10	4/9		5/10	2/10

Age (years)	40.07 ± 21.3	27.92 ± 8.49		30.53 ± 9.3	45.83 ± 18.24

Clinical subtype	NA	CD/UC 8/4		Arthritis positive/negative 4/11	Bone erosion positive/negative 4/8

		CDAI	UCDAI	PASI	DAS28
		
Clinical severity	NA	270.4 ± 67.18	8 ± 1.2	27.47 ± 9.62	6.12 ± 1.05
		
		124-365	6-9	15-48	4.07-7.56

Definition of abbreviations: IBD = Inflammatory bowel disease, CD = Crohn's Disease, CDAI = Crohn's Disease Activity Index, UC = Ulcerative colitis, UC DAI = Ulcerative Colitis Disease Activity Index, PASI = Psoriasis Area Severity Index, DAS28 = Disease Activity Score 28, NA = non available. Data are presented as mean ± SD; also range in Clinical Severity.

### Peripheral blood mononuclear cell collection and RNA processing

Venous peripheral blood samples were collected by clinicians (30 ml) in Venous Blood Vacuum Collection Tubes containing EDTA (BD Vacutainer K2E). PBMCs were separated by Ficoll gradient centrifugation.

Total RNA was extracted from PBMCs using Trizol reagent (Invitrogen), according to the manufacturer's protocol. RNA quality and quantity were checked on NanoDrop and Agilent Bioanalyser 2100 (Agilent Technologies).

### TaqMan mRNA analysis by RT-QPCR

Gene expression data was obtained using TaqMan Low Density Array (TLDA) (Applied Biosystems) which is a 384-well micro fluidic card that enables to perform 384 simultaneous real-time PCR runs and which has been used for gene expression profiling in several studies [13,14]. This low- to medium- throughput micro fluidic card allows for 2 samples to be run in parallel against 96 TaqMan^® ^Gene Expression Assay targets that are pre-loaded into each of the wells on the card. cDNA was generated with High Capacity cDNA Reverse Transcription Kit according to manufacturer's protocol. 2 micrograms of RNA were used per sample in the RT-PCR runs. 400 ng (4 μl) cDNA was used in each sample. 196 μl nuclease-free water and 200 μl 2× TaqMan Universal PCR Master Mix (Applied Biosystems) were added for the Real-Time Quantitative PCR measurements. This mixture was then equally divided over four sample-loading ports of the TLDA, each connected to one set of the 96 genes of interest. The arrays were centrifuged once (1', 1300 RPM on room temperature) to equally distribute the sample over the wells. Subsequently, the card was sealed to prevent an exchange between wells. RT-QPCR amplification was performed using an Applied Biosystems Prism 7900HT sequence detection system with the following thermal cycler conditions: 2 min at 50°C and 10 min at 94.5°C, followed by 40 cycles of 30 s at 97°C and 1 min at 59.7°C.

### Gene List Selection Process

**Figure 1:**
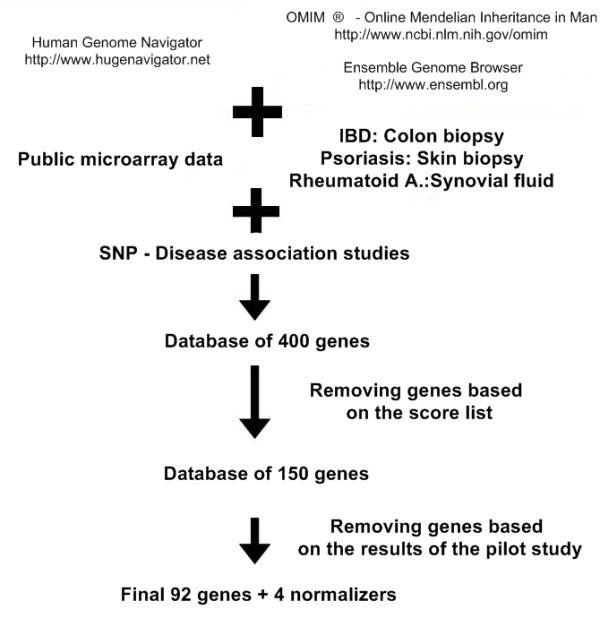
**Flowchart of gene selection process**. A flow chart of the gene selection process shows the steps through which we chose the final 96 genes out of the database containing 400 genes.

A database containing 400 genes which are associated with the 3 chronic inflammatory diseases and inflammation was constructed by using the data derived from the Human Genome Navigator that lists all the genes related to a specific disease and the evidence the relation is based on; the current medical literature and international databases (e.g. OMIM, Entrez Gene). Genes were also selected from genome-wide association studies as well as microarray analyses focusing on skin biopsy in psoriasis, colon biopsy in IBD and synovial fluid in RA. (Figure 1)

In the second step, genes were put in order by using a score list that was based on the number of publications mentioning the disease-gene association, relation to other diseases and expected expression in PBMC according to previous studies and our pilot experiment in which we analyzed selected genes in PBMC samples through individual RT-QPCR assays. This selection process resulted in 150 genes.

In the third step, genes that did not show expression (missing signal in QPCR measurements) in the pilot study were removed, leading to a final list of 96 genes Additional File 1: Figure S1.

### Statistical Analyses

Relative gene expression levels of each gene were calculated by comparative Ct method and results were normalized to glyceraldehyde-3-phosphate dehydrogenase (GAPDH) expression for each sample. Statistical analyses of the normalized gene expression data were performed in Prism (GraphPad). Due to the fact that our data did not follow normal distributions, the gene expression in groups with different numbers of samples was compared separately using the non-parametric Mann-Whitney U test. P-values < 0.05 were considered to be statistically significant. This method that does not include correction for multiple comparisons is widely used in the analysis of TLDA data [15,13] and is explained in details in [16].

Gene interactions were analyzed by using the Direct Interactions and Biological Processes functions of GeneSpring GX (Agilent Technologies).

Principal component analysis (PCA), a standard, non-parametric tool that reduces a complex data set to a lower dimension, was used in order to reveal the internal structure of the data sets and to project the differences between diseased and healthy groups based on each set of significantly changing genes.

## Results

### Identification of gene panels discriminating chronic inflammatory disease patients from healthy controls

**Figure 2:**
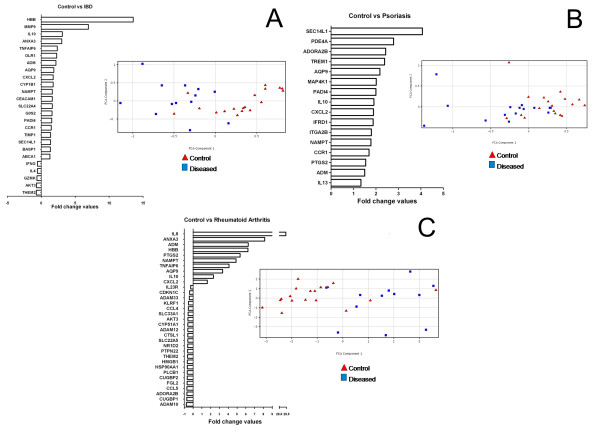
**Fold changes of genes differentiating between diseased and control samples**. Fold change values of genes, showing statistically significant (Mann-Whitney U test) differential expression between diseased and control patients, were generated from RT-QPCR measurements and represent the difference of the means of the diseased and control groups. Principal component analysis was performed and separates the two groups of samples. 2a represents the IBD, 2b the psoriasis and 2c the RA gene panel.

A panel of 96 genes has been selected by the algorithm depicted on Figure 1. Patients were recruited and PBMC gene expression was determined as described in Materials and Methods. When we compared the gene expression results we have found 53 genes that show significant differences between diseased and healthy samples. First, we looked at the gene panels that differentiate each disease from the set of control samples. Altogether 25 genes show significant differences between IBD; 16 genes between psoriasis; 33 genes between RA and controls (Figure 2a-c). Principal component analysis of these gene sets also separates the different groups of samples. In order to get hints of the complex transcriptional basis of inflammatory diseases and to find potential targets that might play a role in the pathogenesis of the conditions, the interaction among multiple genes needs to be revealed. Gene interaction analyses highlighted IFNG, IL4, IL10, MMP9 and TIMP1 in IBD; IL10, IL13 and PTGS2 in psoriasis; IL8, IL10 and PTGS2 in RA. These genes had the most direct interactions that might reveal their key role in the pathogenetic background of the diseases. Gene interaction analysis on each set of genes is provided in the supplement material Additional File 1: Figure S3-5.

### Gene sets showing overlapping or differential expression

**Figure 3:**
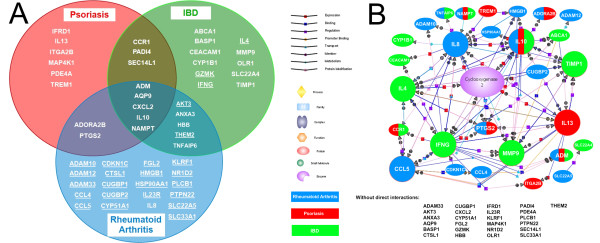
**Genes separating chronic inflammatory diseases from controls**. (a) Venn diagram shows all of those genes that show significant differences between each disease group and control samples. Each set contains genes that separate control samples only from the particular disease or diseases. Underlined genes were down-regulated compared to healthy controls. (b) Gene interaction analysis in GeneSpring GX in Direct Interactions mode highlights 28 genes that have direct interactions with each other while 25 genes have no direct interactions. Genes with the highest number of interactions are shown in extended size. Cyclooxygenase 2 enzyme is located in the middle of this pathway network. The genes that showed significant differences between one of the diseased groups and healthy controls have color codes in which blue represents RA, green represents IBD and red codes for psoriasis-related genes.

In order to analyze the gene expression patterns of these conditions, first we created a Venn diagram highlighting those genes that differentiate between a disease and the set of control samples (Figure 3a).

Performing Gene Interaction Analysis resulted in the identification of a network that highlights the genes with the most interactions such as CCL5, IFNG, IL4, IL8, IL10, IL13, MMP9, PTGS2 and TIMP1 (Figure 3b) clearly showing that not only individual genes but entire networks are impacted and hence can be identified. Although there are genes that showed disease specific signatures, all of these genes showing significant differences between diseased and healthy samples form a network which might represent the common background of the pathogenesis of this type of inflammation.

**Figure 4:**
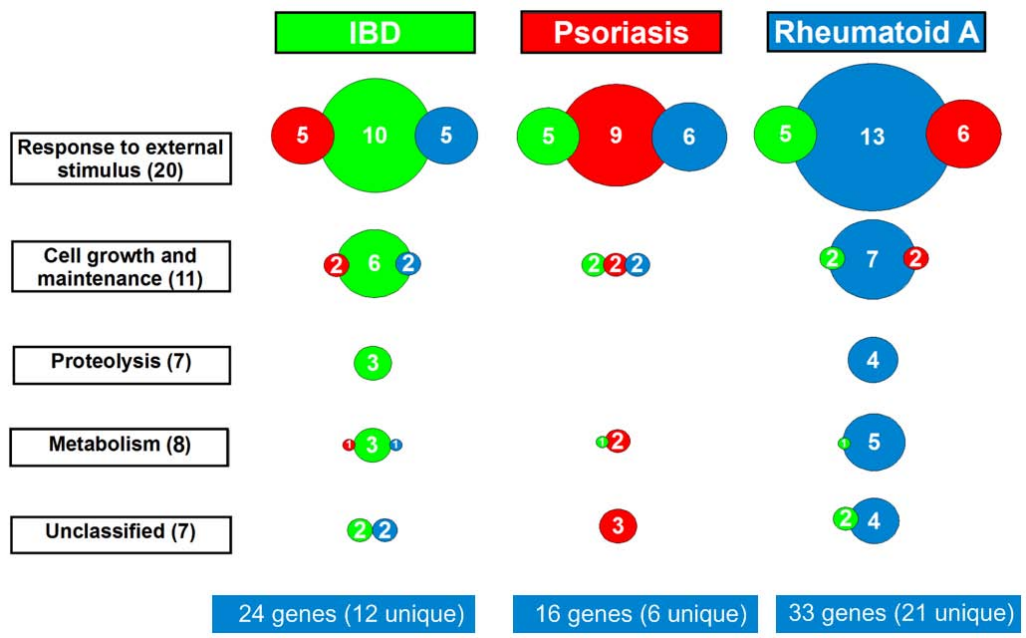
**Functional categorization of significantly changing genes**. We found 53 genes that show significantly differences between chronic inflammatory diseases and control samples. These genes were grouped into functional categories made by EASE. Genes were grouped in the category that had the highest relevance according to the EASE software. Colors represent disease groups. The size of the diagrams correlates with the number of genes in each set. Overlapping sets of genes are shown as overlapping diagrams.

As the next step in our analysis all the significantly changing genes were assigned to functional categories that were created based on EASE, the Expression Analysis Systematic Explorer that provides statistical methods for discovering biological themes within gene lists [17]. Genes were grouped into inflammatory response; cell growth and maintenance; proteolysis; metabolism and unclassified (including genes in unique categories or genes without categories) categories with 21, 11, 7, 9 and 7 genes respectively. In order to illustrate the similarities in the pathogenetic background of these diseases, the functional categories, the number of genes and the disease gene panels are visualized. It shows that there are similar number of genes in the same functional categories in each of the diseases, though the number of unique genes (11 in IBD, 6 in psoriasis and 21 in RA) separating only a disease from the control group is also high (Figure 4).

### Diseases subtype stratification by differentially expressed genes

**Figure 5:**
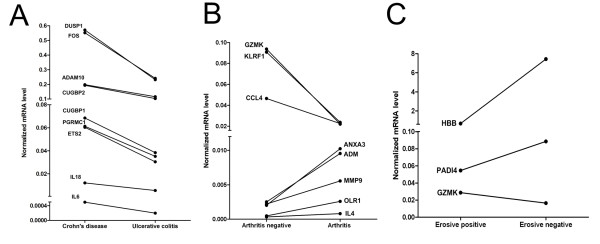
**Correlation between gene expression levels and clinical parameters**. Normalized mRNA levels of genes showing significant differences between patients with (a) Crohn's disease and Ulcerative colitis; (b) psoriasis patients with and without arthritis; (c) RA patients with and without MR-confirmed bone erosion were generated from RT-QPCR measurements and represent the means of the expression levels of the diseased and control groups.

We also sought to identify potential markers that differentiate between distinct subtypes or states of prognosis in each of the three diseases to stratify the disease based on gene expression patterns (Figure 5). Analyses of individual genes are provided in the supplement material Additional File 1: Figure S7-9.

Regarding IBD, ADAM10, CUGBP1, CUGBP2, DUSP1, ETS2, FOS, IL6, IL18 and PGRMC1 show significantly higher expression levels in patients with CD compared to patients with UC (Figure 5a).

In psoriasis, CCL4, GZMK and KLRF1 show significantly higher, while ADM, ANXA3, IL4, MMP9 and OLR1 show significantly lower expression levels in patients with arthritis negative psoriasis compared to patients with symptoms of arthritis (Figure 5b).

HBB and PADI4 show lower; GZMK shows higher expression in RA patients with bone erosions confirmed with Magnetic Resonance Imaging compared to those without such symptoms (Figure 5c).

### Peripheral blood derived universal markers of chronic inflammation

**Figure 6:**
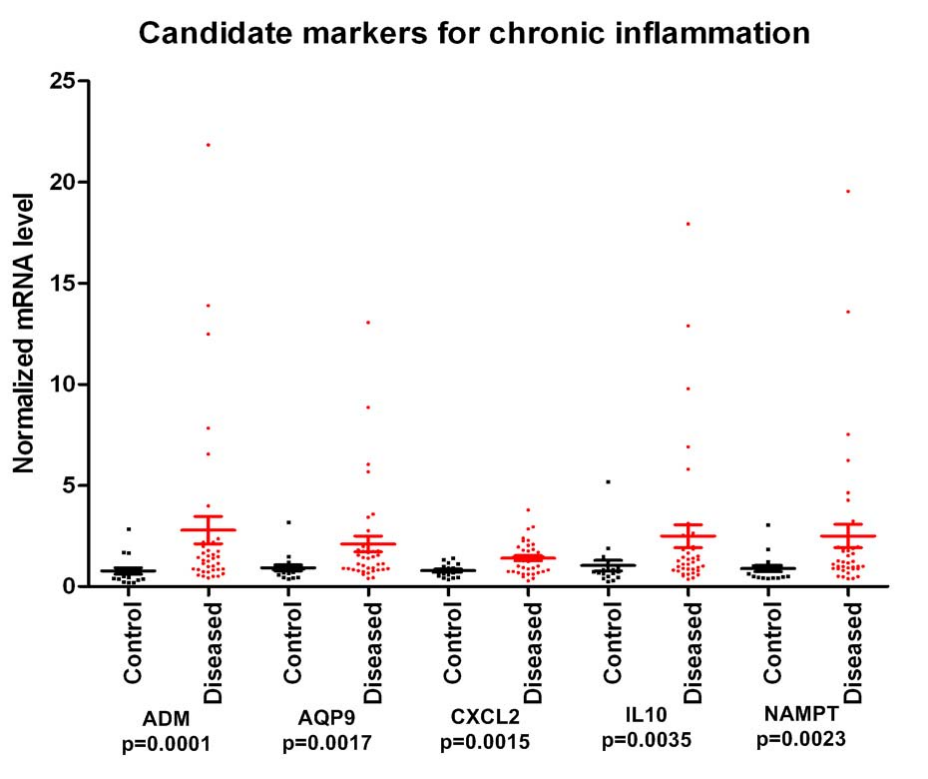
**Peripheral blood derived universal markers of chronic inflammation**. Normalized mRNA levels of genes (with SD) showing significant differences between all the samples of chronic inflammatory diseases and healthy controls were generated from RT-QPCR measurements.

**Figure 7:**
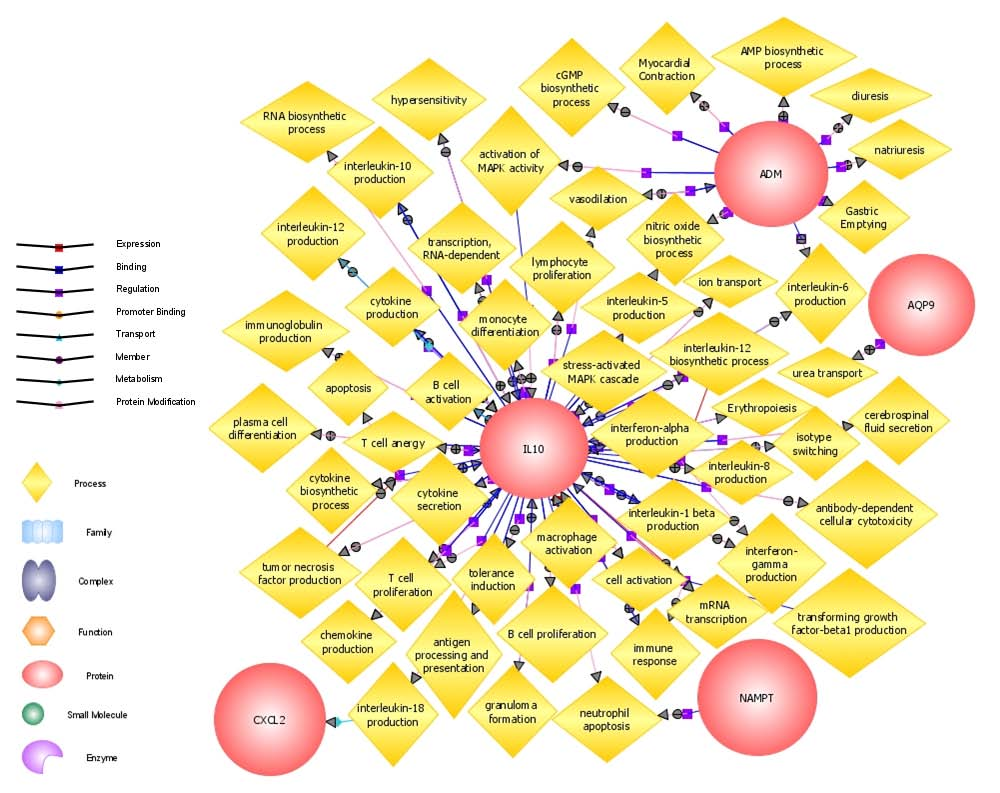
**Pathway analysis of universal markers of chronic inflammation**. Pathway analysis was carried out with GeneSpring GX Biological Processes mode that revealed all the biological processes the genes are related to. Circles represent the 5 genes; deltoids represent biological processes.

Importantly, we have identified five genes, ADM, AQP9, CXCL2, IL10 and NAMPT that show significant differences in expression levels between all the three conditions and control samples (Figure 6). These genes might be considered universal markers of chronic inflammation in PBMCs. Regarding pathway analysis that features the biological processes the genes are associated with; IL10 has the highest number of related biological processes (over 20) including lymphocyte, T and B cell proliferation, interleukin production and macrophage activation. ADM is related to 10 processes such as activation of MAPK-activity and interleukin-6 production; while AQP9, CXCL2 and NAMPT are only associated with a single process, urea transport, interleukin-18 production and neutrophil apoptosis, respectively (Figure 7).

We also determined the number of diseased patients with gene expression levels above the range of the mean + SD or 2 SD of control samples. Additional File 1: Figure S6.

## Discussion

It is of critical importance to identify new biomarkers and hints of mechanisms of disease states and disease progression in illnesses affecting large populations such as chronic inflammatory diseases in order to personalize treatment and accurately monitor disease progression. Methods based on genomic approaches and amongst them global gene expression profiles play an increasing role in such efforts.

The pathomechanism of these chronic inflammatory diseases has been examined at different levels including cell cultures, animal models and clinical samples. Importantly, as the molecular signature of disease across tissues is overall more prominent than the signature of tissue expression across diseases [18], there is good reason to believe one can detect disease-specific gene expression signatures in PBMC samples which are easy-to-access.

There have been studies analyzing the gene expression patterns of these diseases in PBMC samples which led to the identification of genes, which we also found to be significantly different between disease and control patients. Examples include PTGS2 in psoriasis [10]; PTPN22 in RA [19] and GZMK in IBD [12]. Other groups compared different chronic disorders to each others such as juvenile arthritis and spondyloarthropathy [20] or psoriatic arthritis, RA and spondyloarthropathy [21], but such an extended number of genes have not yet been found in peripheral blood. Importantly the gene expression profiles of RA, IBD and psoriasis have not been compared previously. We tried to fill this gap with our study and identify markers of chronic inflammation that overlap among the 3 conditions and also ones that discriminate them from each other.

It should be noted though gene expression analyses of these diseases provide little insight into the pathomechanisms of the disease simply because it is impossible to sort out what is cause and what is consequence. However it can still provide hints or clues about the potential pathways affected. Therefore such datasets, although indirectly, can form the basis of more mechanistic studies. This is why we have carried out and presented the gene interaction analyses of the genes identified and linked to the different diseases. Significantly the existence of overlapping gene sets suggests that there are common pathways impacted among the studied diseases. A common characteristic of all diseases is an altered inflammatory response.

In addition, genetic association studies have already identified SNPs linked to these diseases. A comparison of our dataset to these can provide further support to those findings [Table 1].

Several of the now identified differentially expressed genes have been linked to the studied diseases although either at a different level or different model. Regarding the IBD gene panel, OLR1/LOX1 [22] and CCR1 [23] both have immune response function and are expressed in human intestinal cells. The homophilic ligation of CEACAM1, which is a cell-surface molecule, on T cells leads to the inhibition of a range of T-cell functions therefore it might be a new potential therapeutic target in the treatment of IBD [24]. MMP9, a gelatinase, that showed a 7-fold up-regulation in diseased samples in our study is an important mediator of tissue injury in colitis and is up-regulated in sputum samples of CD patients [25]. Garg et al. suggested that developing strategies to block MMP-9 activity in the gut might be of benefit to IBD [26]. TIMP1 is a natural inhibitor of matrix metalloproteinases (MMPs) and might be considered a potential drug target. The DNA methylation of IFNG that regulates immune response was analyzed within the mucosal compartment in both normal and IBD populations [27]. PTGS2/COX2 is related to IBD expression level in colon biopsy [28]. ABCA1, BASP1, GZMK, HBB, SEC14L1, THEM2 and TNFAIP6 have never been associated with IBD; therefore further analyses should be carried out.

In the psoriasis panel, CCR1 that plays a role in inflammatory response is associated with psoriasis via the analysis of skin lesion samples [29]. Koczan et al. identified pairs of genes such as PTGS2/COX2 and NR1D2 which allowed an accuracy of disease stage prediction of 86%, based on gene expression patterns [10]. ADORA2B, IFRD1, ITGA2B, MAP4K1, PADI4, PDE4A, SEC14L1 and TREM1 have no documented association with psoriasis yet. Therefore these are candidates for further SNP/genetic association or mechanistic studies.

Regarding the significantly changing genes in RA samples, IL8 is related to RA based on expression [30]. Van Der Voort et al. demonstrated that the expression of ADAM10 is strongly enhanced in RA synovia [31]. CCL4 was expressed in PBMC [30] and CCL5 showed high serum levels [32]. Kehlen et al. found up-regulation of TNFAIP6 in fibroblast-like synoviocytes of patients with RA [33]. HMGB1 [34] and PLCB1 [35] only showed difference in protein level in RA patients. PTGS2/COX2 mRNA levels in PBMC samples from RA patients were within the normal range or below normal [36]. ADAM12, ADAM33, ADORA2B, AKT3, ANXA3, CDKN1C, CTSL1, CUGBP1, CUGBP2, CYP51A1, FGL2, HBB, HSP90AA1, KLRF1, NR1D2, SLC33A1 and THEM2 have no evidence of association with RA and should further be studied.

Gene expression profiling may allow early diagnosis, aid in identifying prognosis or subtypes. We found genes that differentiate between CD and UC in IBD of which some have already been associated with the disease but at a different level or in a different tissue. Alexander et al. reported that the expression level of FOS was two- to threefold higher in involved than in uninvolved areas of the colons of two UC patients [37]. van der Pouw Kraan at el. found that ETS2 may be an important transcription factor driving inflammation in acute as well as chronic inflammatory diseases such as IBD [38]. As oppose to UC, IL-18 may serve as indicators of acute phase reactivity in CD, according to Haas et al. [39], that correlates with our findings. Mitsuyama et al. suggested that IL-6 trans-signaling may play role in the development of IBD and imply the possibility of a selective therapeutic strategy to target this signaling [40].

A gene panel containing 8 genes shows significant differences between patients with and without arthritis in psoriasis. Hitchon et al. described that MMPs, especially MMP2 and MMP9, have been implicated in several features of inflammatory arthritis including angiogenesis and bone erosions [41]. Partsch et al. measured the protein level of IL4 in synovial fluid of patients with psoriatic arthritis (PsA) [42].

Another gene set including HBB, GZMK and PADI4 separates different states of prognosis in RA regarding MR-confirmed bone erosion. In RA, PADI4 is a target of autoantibodies; and its increased expression in synovia of RA patients [43], also its relation to the disease at SNP level have already been confirmed [44]. Osteoclasts are shown to be mainly involved in the bone-destruction of RA which may indicate further studies regarding the role of PADI4 in bone erosion.

We consider a key aspect of our work is the identification of five genes including ADM, AQP9, CXCL2, IL10 and NAMPT that differentiate between samples from patients with chronic inflammation and healthy controls. ADM that plays role in response to wounding is found to be distributed on the surface of the human colonic mucosa [45] and the plasma level of ADM in RA patients was significantly higher compared to healthy controls in synovial tissue [46]. CXCL2 that has function in immune response is over-expressed in osteoarthritis fibroblasts rather than rheumatoid fibroblasts [47], up-regulated in psoriatic epidermis [48], and also in epithelial tissue [49]. IL10 is expressed in PBMCs of psoriasis [50], RA [51] and IBD patients [52]. The expression of NAMPT which is a pre-B cell-enhancing factor is increased in colonic biopsy specimens of IBD patients compared to healthy controls [53], it is also up-regulated in plasma and synovial fluid of RA patients [54]; and in PBMC of psoriasis in the diseased stage [10]. AQP9 that has an activity in immune response has never been associated with any of these medical conditions.

These genes might serve as universal markers of chronic inflammation. As expected, IL10 seems to be the key gene in this network regarding biological processes (Figure 3b). As the number of biological processes related to ADM is relatively high and it has no direct interactions with the genes we examined, there might be other potential markers that play role in the pathomechanism of the conditions we analyzed that also have similar pathways or targets as ADM. Chronic inflammatory and autoimmune diseases share a number of phenotypic and genetic characteristics suggesting common etiological pathways or pathomechanisms. Becker et al. used meta-analyses of whole-genome scans and found non-random clustering of disease susceptibility loci for autoimmune diseases [55]. Our results may suggest that all of the chronic inflammatory conditions we analyzed share similar pathogenetic background as reflected by peripheral gene expression.

Personalized medicine is becoming an integral part of healthcare and the key challenge is to establish a strategic focus on biomarker-based clinical tests. Non-invasive or minimally invasive diagnostic tests present less danger to a patient than invasive tests such as biopsies; and can help clinicians by providing them with valuable information in decision-making. A biomarker assay based on the gene panels described above might save time by reducing a list of preliminary disease impressions to a definitive diagnosis. The evaluation of these results and the identification of genes with altered expression or marker genes could also be potential targets for novel and more effective therapies and may lead to important insights into the pathogenesis of chronic inflammatory diseases. It might also have benefits to look at the identified gene panels in terms of SNPs in order to identify potential genetic changes associated with these medical conditions. Such novel and known biomarkers or a panel of such markers can play a major role in the development of personalized medicine.

## Conclusion

Our results suggest that IBD, psoriasis and rheumatoid arthritis have common pathogenetic background and gene expression profiling from peripheral blood might reveal novel targets and pathways affected by these diseases.

## Abbreviations

ABCA1: ATP-binding cassette, sub-family A, member 1; ADAM10: ADAM metallopeptidase domain 10; ADAM12: ADAM metallopeptidase domain 12; ADAM33: ADAM metallopeptidase domain 33; ADM: Adrenomedullin; ADORA2B: adenosine A2b receptor; AKT3: v-akt murine thymoma viral oncogene homolog 3; ANXA3: annexin A3; AQP9: Aquaporin 9; BASP1: brain abundant, membrane attached signal protein 1; CCL4: chemokine (C-C motif) ligand 4; CCL5: chemokine (C-C motif) ligand 5; CCR1: chemokine (C-C motif) receptor 1; CDKN1C: cyclin-dependent kinase inhibitor 1C; CEACAM1: carcinoembryonic antigen-related cell adhesion molecule 1; CTSL1: cathepsin L1; CUGBP1: CUG triplet repeat, RNA binding protein 1; CUGBP2: CUG triplet repeat, RNA binding protein 2; CXCL2: chemokine (C-X-C motif) ligand 2; CYP1B1: cytochrome P450, family 1, subfamily B, polypeptide 1; CYP51A1: cytochrome P450, family 51, subfamily A, polypeptide 1; FGL2: fibrinogen-like 2; GK: glycerol kinase; GZMK: granzyme K; HBB: hemoglobin, beta; HSP90AA1: heat shock protein 90 kDa alpha (cytosolic), class A member 1; IBD: Inflammatory bowel disease; IFNG: interferon gamma; IFRD1: interferon-related developmental regulator 1; IL10: Interleukin 10; IL13: Interleukin 13; IL23R: Interleukin 23 receptor; IL4: Interleukin 4; IL8: Interleukin 8; ITGA2B: integrin, alpha 2b; KLRF1: killer cell lectin-like receptor subfamily F, member 1; MAP4K1: mitogen-activated protein kinase 1; MMP9: matrix metallopeptidase 9; NAMPT: nicotinamide phosphoribosyltransferase; NR1D2: nuclear receptor subfamily 1, group D, member 2; OLR1: oxidized low density lipoprotein receptor 1; PADI4: peptidyl arginine deiminase, type IV; PBMC: Peripheral Blood Mononuclear Cells; PDE4A: cAMP-specific phosphodiesterase 4D; PLCB1: phospholipase C, beta 1 (phosphoinositide-specific); PTGS2: prostaglandin-endoperoxide synthase 2 (prostaglandin G/H synthase and cyclooxygenase); PTPN22: protein tyrosine phosphatase, non-receptor type 22 (lymphoid); QPCR: Quantitative polymerase chain reaction; RA: Rheumatoid arthritis; SEC14L1: SEC14-like 1 (S. cerevisiae); SLC22A4: solute carrier family 22 member 4; SLC22A5: solute carrier family 22 member 5; SLC33A1: solute carrier family 33 member 1; THEM2: acyl-CoA thioesterase 13; TIMP1: TIMP metallopeptidase inhibitor 1; TLDA: Taqman Low Density Array; TNFAIP6: tumor necrosis factor, alpha-induced protein 6; TREM1: triggering receptor expressed on myeloid cells 1

## Competing interests

The authors declare that they have no competing interests.

## Authors' contributions

B.M designed and carried out experiments, analyzed data and wrote the paper. S.P. designed and carried out experiments and analyzed the data, A.S., Z.S., K.P. and M.P. determined patient inclusion and exclusion criteria, carried out patient recruitment and collection. L.N. directed research, designed experiments, analyzed the data and wrote the paper.

## Pre-publication history

The pre-publication history for this paper can be accessed here:

http://www.biomedcentral.com/1755-8794/3/15/prepub

## Supplementary Material

Additional file 1 **List of genes, gene interaction analyses and detailed gene expression data**. • Figure S1) List of genes that were measured on the TaqMan Low Density Arrays. • Figure S2) Inclusion and exclusion criteria of diseased patients and healthy controls. • Figure S3) Gene interaction analysis ("Cell mode" in GeneSpring GX) of the genes differentiating between IBD and controls. • Figure S4) Gene interaction analysis ("Cell mode" in GeneSpring GX) of the genes differentiating between psoriasis and controls. • Figure S5) Gene interaction analysis ("Cell mode" in GeneSpring GX) of the genes differentiating between RA and controls. • Figure S6) Number of diseased patients with gene expression levels outside the range of the mean ± SD or 2 SD in control sample. Based on Figure 6. • Figure S7) Stratifying IBD: The results of the RT-QPCR measurements for each gene that showed significant differences between Crohn's disease vs Ulcerative colitis patients. • Figure S8) Stratifying Psoriasis: The results of the RT-QPCR measurements for each gene that showed significant differences between arthritis negative vs positive forms. • Figure S9) Stratifying RA: The results of the RT-QPCR measurements for each gene that showed significant differences between patients with MRI confirmed bone erosion vs patients without bone erosion. Click here for file
