# Supplementary material for: Peripheral blood gene expression patterns discriminate among chronic inflammatory diseases and healthy controls and identify novel targets
Source: BMC Med Genomics. 2010 May 5;3:15. doi: 10.1186/1755-8794-3-15 (PMC2874757; doi:10.1186/1755-8794-3-15)

**Supplemental Figure S1**

List of genes (with gene name and Applied Biosystems ID) that were included in the Taqman Low Density Arrays.

| | Gene name | ABI ID |  |  | Gene name | ABI ID | | --- | --- | --- | --- | --- | --- | | ABCA1 | Hs01059122_m1 |  |  | IL10 | Hs00174086_m1 | | ACTB | Hs99999903_m1 |  |  | IL13 | Hs00174379_m1 | | DAM10 | Hs00153853_m1 |  |  | IL17A | Hs00174383_m1 | | ADAM12 | Hs01106104_m1 |  |  | IL18 | Hs00155517_m1 | | ADAM33 | Hs00905552_m1 |  |  | IL1B | Hs00174097_m1 | | ADM | Hs00181605_m1 |  |  | IL1RN | Hs00893626_m1 | | ADORA2B | Hs00386497_m1 |  |  | IL23R | Hs00332759_m1 | | ADORA3 | Hs00181232_m1 |  |  | IL2 | Hs00174114_m1 | | AKT3 | Hs00178533_m1 |  |  | IL4 | Hs00174122_m1 | | ALOX5 | Hs00167536_m1 |  |  | IL6 | Hs00174131_m1 | | ANXA3 | Hs00971411_m1 |  |  | IL8 | Hs00174103_m1 | | AQP9 | Hs00175573_m1 |  |  | ITGA2B | Hs01116228_m1 | | BASP1 | Hs00234720_g1 |  |  | KLF6 | Hs00810569_m1 | | CCL4 | Hs99999148_m1 |  |  | KLRF1 | Hs00212979_m1 | | CCL5 | Hs00174575_m1 |  |  | MAP4K1 | Hs00179345_m1 | | CCR1 | Hs00174298_m1 |  |  | MEGF9 | Hs00391048_m1 | | CDKN1A | Hs00355782_m1 |  |  | MMP9 | Hs00234579_m1 | | CDKN1C | Hs00175938_m1 |  |  | MYLIP | Hs00203131_m1 | | CEACAM1 | Hs00236077_m1 |  |  | NAMPT | Hs00237184_m1 | | CLU | Hs00156548_m1 |  |  | NR1D2 | Hs00233309_m1 | | CTSL1 | Hs00266474_m1 |  |  | OLR1 | Hs00234028_m1 | | CUGBP1 | Hs00198069_m1 |  |  | PADI4 | Hs00202612_m1 | | CUGBP2 | Hs00272516_m1 |  |  | PDE4A | Hs00183479_m1 | | CXCL2 | Hs00236966_m1 |  |  | PDE4B | Hs00277080_m1 | | CXCL3 | Hs00171061_m1 |  |  | PGRMC1 | Hs00198499_m1 | | CYLD | Hs00211000_m1 |  |  | PHLDA1 | Hs00705810_s1 | | CYP1B1 | Hs00164383_m1 |  |  | PHLDA2 | Hs00169368_m1 | | CYP51A1 | Hs00426415_m1 |  |  | PLCB1 | Hs00248563_m1 | | DUSP1 | Hs00610257_g1 |  |  | PPIA | Hs99999904_m1 | | EMP1 | Hs00608055_m1 |  |  | PTGS2 | Hs00153133_m1 | | EMR3 | Hs00261470_m1 |  |  | PTPN22 | Hs00249262_m1 | | ETS2 | Hs01036300_g1 |  |  | RPLP0 | Hs99999902_m1 | | FGL2 | Hs00173847_m1 |  |  | RUNX1 | Hs00231079_m1 | | FN1 | Hs01549940_m1 |  |  | S100A11 | Hs00271612_m1 | | FOS | Hs00170630_m1 |  |  | SEC14L1 | Hs00608163_m1 | | FPR1 | Hs00181830_m1 |  |  | SERPINB2 | Hs00234032_m1 | | G0S2 | Hs00274783_s1 |  |  | SLC22A4 | Hs00268200_m1 | | GAPDH | Hs99999905_m1 |  |  | SLC22A5 | Hs00929869_m1 | | GK | Hs02340011_g1 |  |  | SLC33A1 | Hs00270469_m1 | | GZMK | Hs00157878_m1 |  |  | SPP1 | Hs00167093_m1 | | HBB | Hs00758889_s1 |  |  | TCL1A | Hs00951350_m1 | | HES1 | Hs00172878_m1 |  |  | THBS1 | Hs00170236_m1 | | HMGB1 | Hs01923466_g1 |  |  | THEM2 | Hs00218448_m1 | | HSP90AA1 | Hs00743767_sH |  |  | TIMP1 | Hs99999139_m1 | | ICAM1 | Hs99999152_m1 |  |  | TLR2 | Hs00152932_m1 | | IFNG | Hs99999041_m1 |  |  | TNFAIP6 | Hs00200180_m1 | | IFRD1 | Hs00155477_m1 |  |  | TREM1 | Hs00218624_m1 | |  |  |  |  | VEGFA | Hs00903129_m1 | |  |  |
| --- | --- | --- | --- | --- | --- | --- | --- | --- | --- | --- | --- | --- | --- | --- | --- | --- | --- | --- | --- | --- | --- | --- | --- | --- | --- | --- | --- | --- | --- | --- | --- | --- | --- | --- | --- | --- | --- | --- | --- | --- | --- | --- | --- | --- | --- | --- | --- | --- | --- | --- | --- | --- | --- | --- | --- | --- | --- | --- | --- | --- | --- | --- | --- | --- | --- | --- | --- | --- | --- | --- | --- | --- | --- | --- | --- | --- | --- | --- | --- | --- | --- | --- | --- | --- | --- | --- | --- | --- | --- | --- | --- | --- | --- | --- | --- | --- | --- | --- | --- | --- | --- | --- | --- | --- | --- | --- | --- | --- | --- | --- | --- | --- | --- | --- | --- | --- | --- | --- | --- | --- | --- | --- | --- | --- | --- | --- | --- | --- | --- | --- | --- | --- | --- | --- | --- | --- | --- | --- | --- | --- | --- | --- | --- | --- | --- | --- | --- | --- | --- | --- | --- | --- | --- | --- | --- | --- | --- | --- | --- | --- | --- | --- | --- | --- | --- | --- | --- | --- | --- | --- | --- | --- | --- | --- | --- | --- | --- | --- | --- | --- | --- | --- | --- | --- | --- | --- | --- | --- | --- | --- | --- | --- | --- | --- | --- | --- | --- | --- | --- | --- | --- | --- | --- | --- | --- | --- | --- | --- | --- | --- | --- | --- | --- | --- | --- | --- | --- | --- | --- | --- | --- | --- | --- | --- | --- | --- | --- | --- | --- | --- | --- | --- | --- | --- | --- | --- | --- | --- | --- | --- | --- | --- | --- | --- | --- | --- | --- | --- | --- | --- | --- | --- | --- | --- | --- | --- | --- | --- | --- | --- | --- | --- | --- | --- | --- | --- | --- | --- | --- | --- | --- | --- | --- | --- | --- | --- | --- | --- | --- | --- | --- | --- | --- | --- | --- | --- | --- | --- | --- | --- | --- | --- | --- | --- | --- | --- |
|  |  | |
|  |  | |
|  |  | |
|  |  | |
|  |  | |
|  |  | |
|  |  | |
|  |  | |
|  |  | |
|  |  | |
|  |  | |
|  |  | |
|  |  | |
|  |  | |
|  |  | |
|  |  | |
|  |  | |
|  |  | |
|  |  | |
|  |  | |
|  |  | |
|  |  | |
|  |  | |
|  |  | |
|  |  | |
|  |  | |
|  |  | |
|  |  | |
|  |  | |
|  |  | |
|  |  | |
|  |  | |
|  |  | |
|  |  | |
|  |  | |
|  |  | |
|  |  | |
|  |  | |
|  |  | |
|  |  | |
|  |  | |
|  |  | |

**Supplemental Figure S2**

Inclusion and exclusion criteria of patients in each diseases and control group.

|  | **Inclusion criteria** | **Exclusion criteria** |
| --- | --- | --- |
| IBD patients | Clinical diagnosis of Crohn's disease or ulcerative colitis | Pregnant or breastfeeding women |
|  | Age between 15 and 40 | Current or previously known malignome |
|  | Newly diagnosed, treatment-free patients | Clinically essential co-morbidity |
| Psoriasis patients | Active psoriasis (chronic plaque form) | Pregnant or breastfeeding women |
|  | PASI score >10 | Current or previously known malignome |
|  | Age between 15-70 | Clinically essential co-morbidity |
|  | Haven't received systemic therapy for at least 4 weeks (retinoids, methotrexate, cyclosporin, azathioprin) or light therapy |  |
|  |  |  |
|  |  |  |
|  | Have no other inflammation |  |
|  | Have no other skin symptoms |  |
| Rheumatoid arthritis patients | Clinical diagnosis of rheumatoid arthritis | Pregnant or breastfeeding women |
|  | Age between 25 and 70 |  |
|  | Haven't received systemic therapy for at least 4 weeks (antiallergic drugs or immunosupressant agents) | Current or previously known malignome |
|  |  | Clinically essential co-morbidity |
|  | DAS28 (Disease Activity Score) > 5.1 |  |
| Healthy controls | Sex and age-matched patients | Pregnant or breastfeeding women |
|  | Have no inflammation |  |
|  | Haven't received anti-inflammmatory drugs for at least 4 weeks | Current or previously known malignome |
|  |  | Clinically essential co-morbidity |

**Supplemental Figure S3**

Gene interaction analysis (“Cell mode” in GeneSpring GX) of the genes differentiating between IBD and controls.


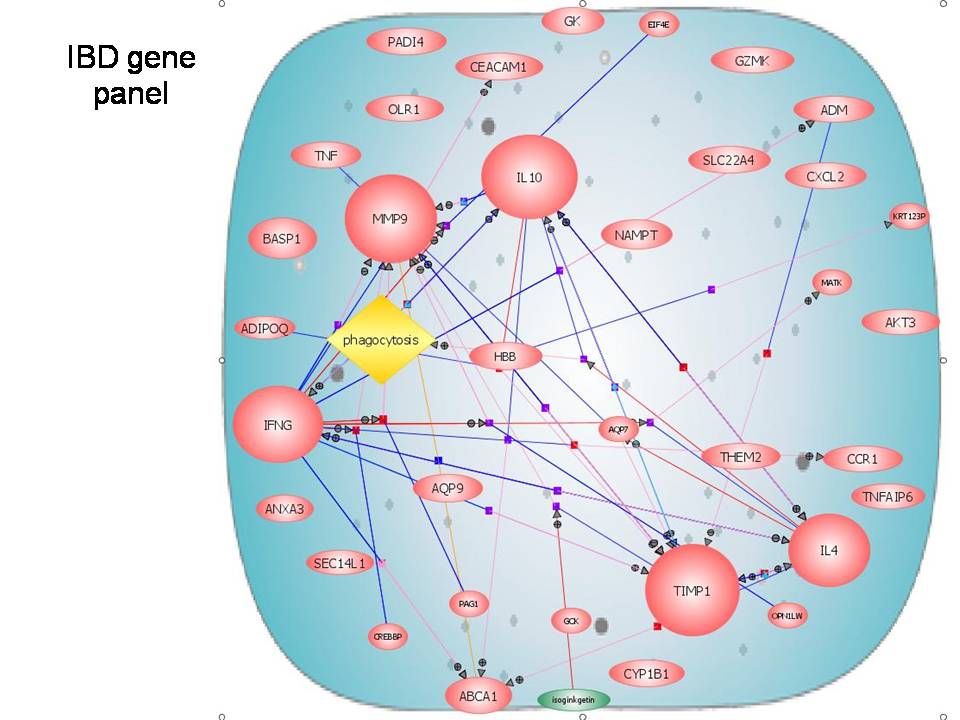


Figure legends:


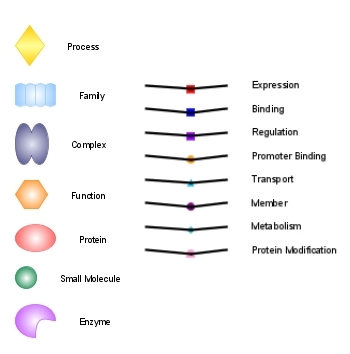


**Supplemental Figure S4**

Gene interaction analysis (“Cell mode” in GeneSpring GX) of the genes differentiating between Psoriasis and controls.


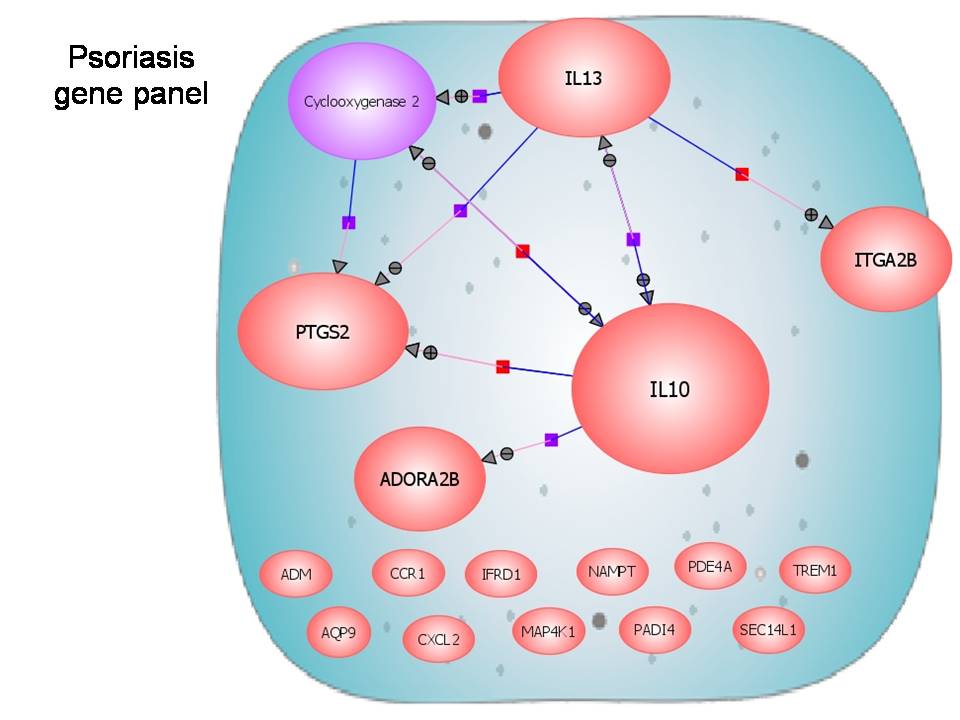


Figure legends:


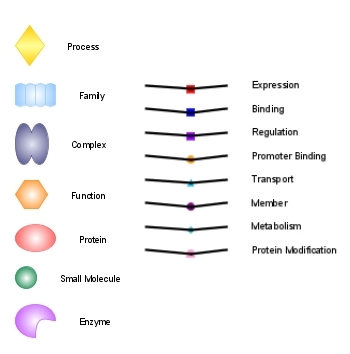


**Supplemental Figure S5**

Gene interaction analysis (“Cell mode” in GeneSpring GX) of the genes differentiating between Rheumatoid arthritis and controls.


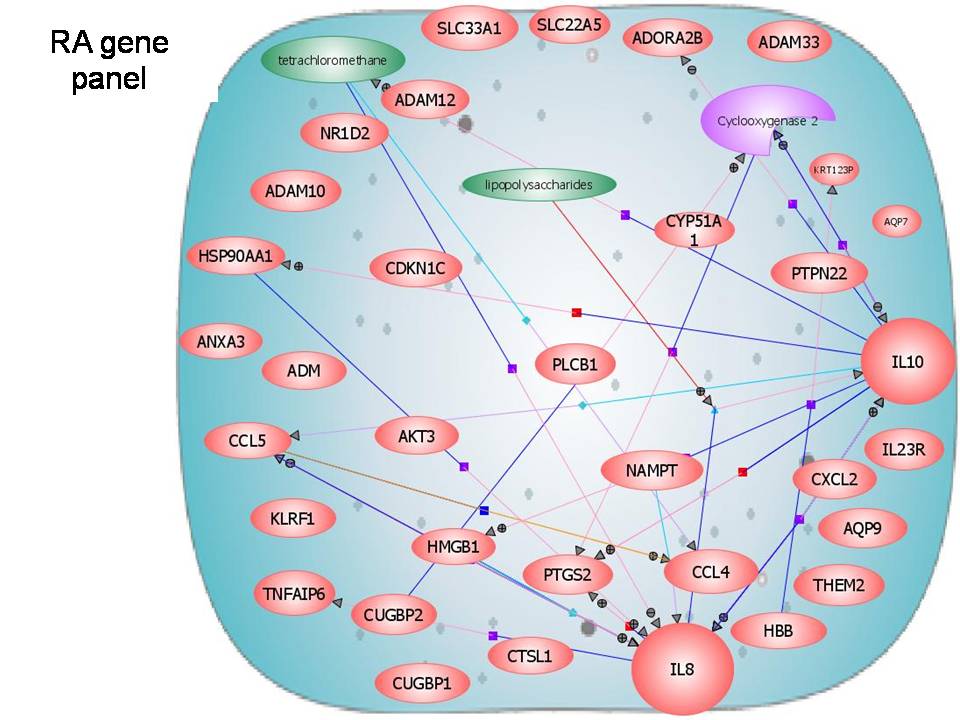


Figure legends:


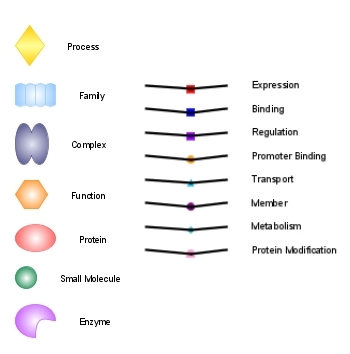


**Supplemental Figure S6**

Number of diseased patients with gene expression levels outside the range of the mean ± SD or 2 SD in control samples. Based on Figure 6.

|  | **ADM** | **AQP9** | **CXCL2** | **IL10** | **NAMPT** |
| --- | --- | --- | --- | --- | --- |
| Mean + SD in control samples | 1.41 | 1.53 | 1.08 | 2.11 | 1.51 |
| Number of diseased patients above this range | 17 | 18 | 23 | 10 | 18 |
| Mean + 2SD in control samples | 2.07 | 2.14 | 1.4 | 3.19 | 2.16 |
| Number of diseased patients above this range | 12 | 9 | 18 | 5 | 9 |

**Supplemental Figure S7**

Stratifying IBD: The results of the RT-QPCR measurements for each gene that showed significant differences between Crohn’s disease vs Ulcerative colitis patients.


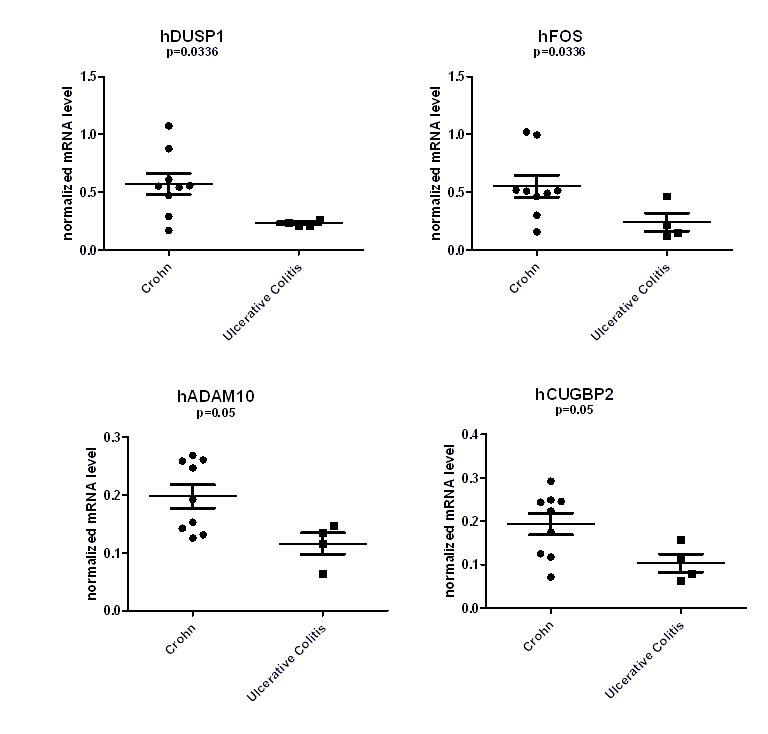


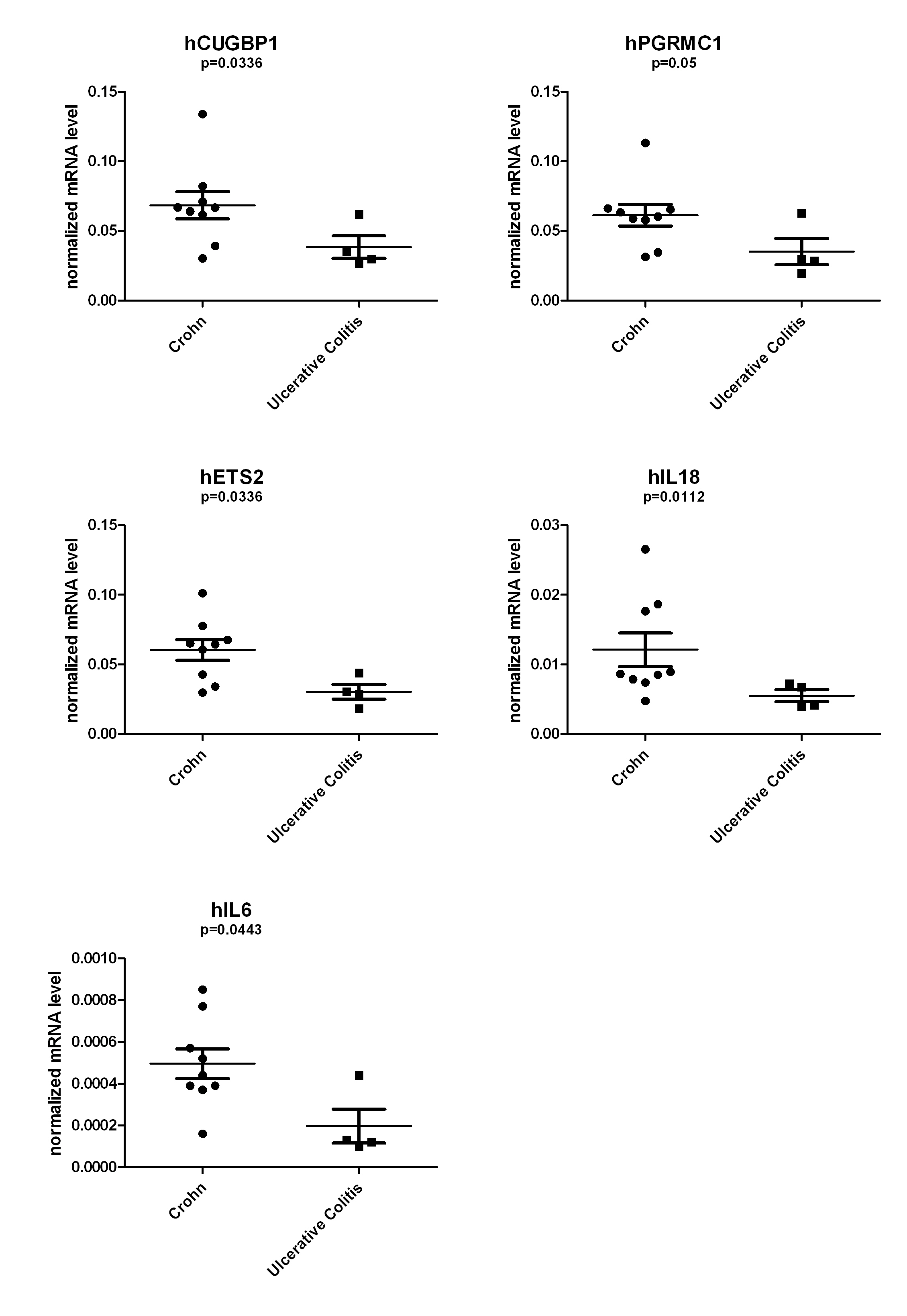


**Supplemental Figure S8**

Stratifying Psoriasis: The results of the RT-QPCR measurements for each gene that showed significant differences between arthritis negative vs positive forms.


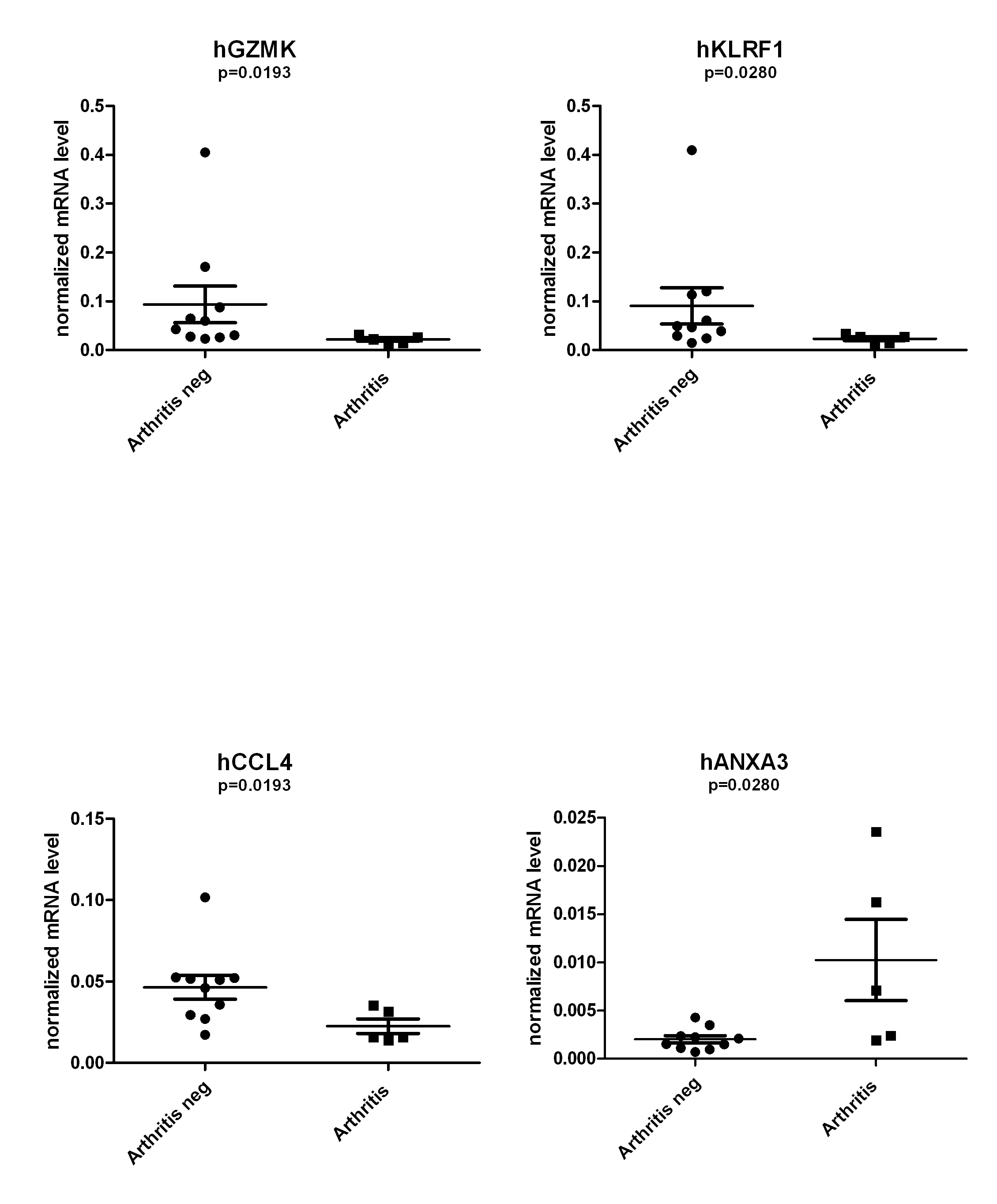


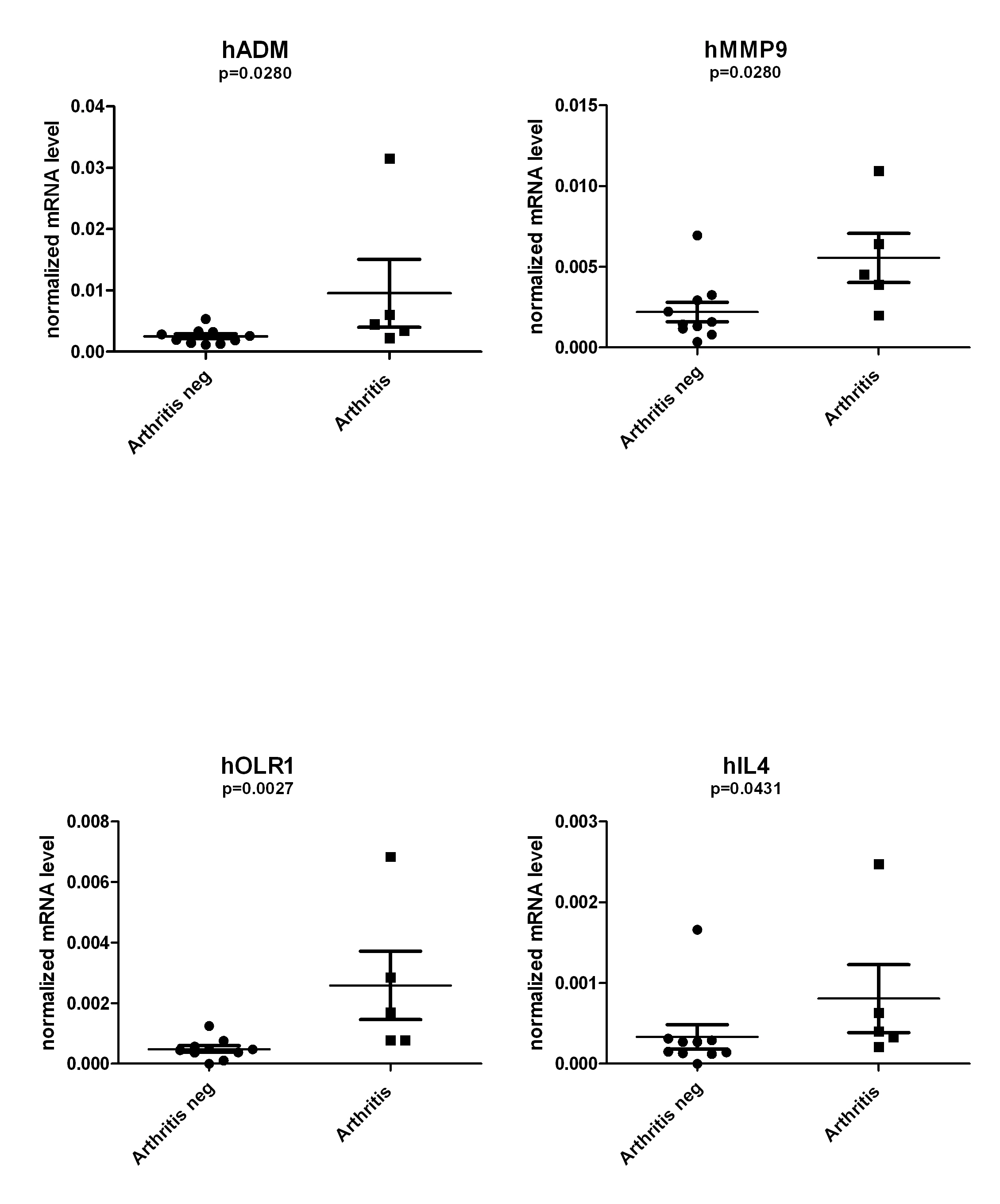


**Supplemental Figure S9**

Stratifying RA: The results of the RT-QPCR measurements for each gene that showed significant differences between patients with MRI confirmed bone erosion vs patients without bone erosion.

.
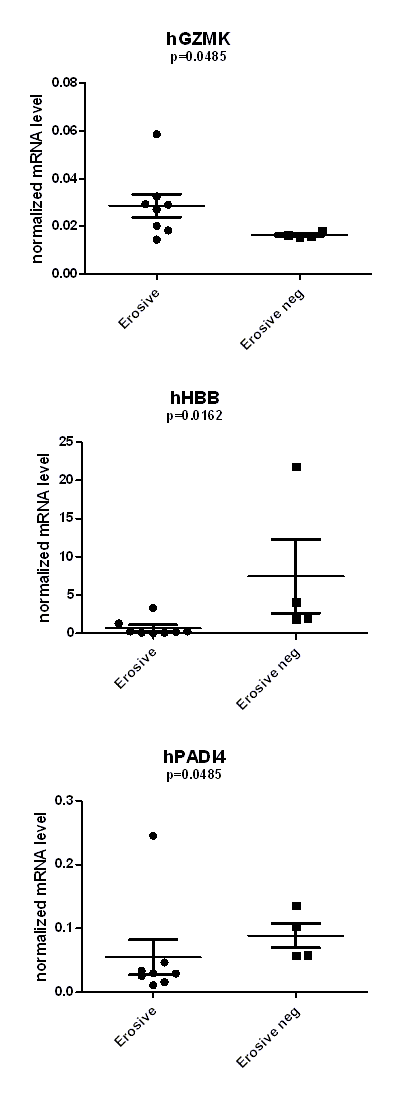

Supplement: Additional file 1 — List of genes, gene interaction analyses and detailed gene expression data. • Figure S1) List of genes that were measured on the TaqMan Low Density Arrays. • Figure S2) Inclusion and exclusion criteria of diseased patients and healthy controls. • Figure S3) Gene interaction analysis ("Cell mode" in GeneSpring GX) of the genes differentiating between IBD and controls. • Figure S4) Gene interaction analysis ("Cell mode" in GeneSpring GX) of the genes differentiating between psoriasis and controls. • Figure S5) Gene interaction analysis ("Cell mode" in GeneSpring GX) of the genes differentiating between RA and controls. • Figure S6) Number of diseased patients with gene expression levels outside the range of the mean ± SD or 2 SD in control sample. Based on Figure 6. • Figure S7) Stratifying IBD: The results of the RT-QPCR measurements for each gene that showed significant differences between Crohn's disease vs Ulcerative colitis patients. • Figure S8) Stratifying Psoriasis: The results of the RT-QPCR measurements for each gene that showed significant differences between arthritis negative vs positive forms. • Figure S9) Stratifying RA: The results of the RT-QPCR measurements for each gene that showed significant differences between patients with MRI confirmed bone erosion vs patients without bone erosion. [file 1755-8794-3-15-S1.DOC]
